# The Next Chapter in TAVR: Innovations and the Road Ahead

**DOI:** 10.3390/jcm14134504

**Published:** 2025-06-25

**Authors:** Philippe Brouillard, El Hadji Diallo, Walid Ben Ali, Rémi Kouz

**Affiliations:** 1 Département de Médecine, Université de Montréal, Montréal, QC H3C 3J7, Canada; 2 Institut de Cardiologie de Montréal, Montréal, QC H1T 1C8, Canada; 3 Hôpital du Sacré-Cœur de Montréal, Montréal, QC H4J 1C5, Canada

**Keywords:** lifetime management, TAVR, innovation, structural interventional cardiology, aortic valve stenosis

## Abstract

Transcatheter aortic valve replacement (TAVR) was first introduced as a minimally invasive treatment for patients with severe aortic stenosis (AS) who are at high or intermediate surgical risk. Recently, its application has expanded to include younger and lower-risk patients, establishing TAVR as a less invasive alternative to surgical aortic valve replacement (SAVR) across the entire surgical spectrum. The expanding utilization of TAVR has driven significant advancements that have greatly enhanced its safety and effectiveness, resulting in a substantial reduction in complications such as paravalvular leak, conduction abnormalities, and periprocedural strokes. Numerous trials have demonstrated the potential superiority of TAVR over conventional surgery in achieving favorable clinical outcomes. Furthermore, the increasing number of long-term trials has provided valuable insight into TAVR outcomes in previously under-studied populations, including patients with complex anatomies. However, significant challenges remain, particularly in ensuring the long-term durability of transcatheter valves, with younger patients likely to outlive their bioprosthetic valves. Consequently, the focus is shifting towards lifetime management strategies, including considerations for coronary re-access, the risk of coronary obstruction, and prosthesis–patient mismatch. This review explores key developments in the field, including TAVR for aortic regurgitation and bicuspid anatomy, the emerging role of TAVR in moderate and asymptomatic AS, and innovations in valve design and procedural planning. We also examine novel imaging tools, adjunctive technologies, and strategies to address coronary access and re-intervention. As long-term data accumulate, these evolving trends will shape the future of TAVR and its role in managing aortic valve disease across increasingly complex clinical scenarios.

## 1. Background

Aortic stenosis (AS) is the most common valvular heart disease in adults, particularly in older populations [1], and poses a growing burden as the population ages. When left untreated, severe AS carries high morbidity and mortality [2], resulting in substantial economic costs and increased pressure on healthcare systems [3]. Since the first transcatheter aortic valve replacement (TAVR) in 2002 [4], the procedure has rapidly evolved into a safe and effective treatment, now extending beyond high-risk patients to broader populations. This review explores recent and anticipated developments that define the next chapter of TAVR. These include (1) expanding indications such as aortic regurgitation (AR), bicuspid aortic valve (BAV) disease, and moderate or asymptomatic AS; (2) advances in pre-procedural imaging and patient selection, including the integration of cardiac computed tomography (CT) and machine learning-based simulation tools; (3) innovations in post-procedural coronary access; (4) the increasing use of adjunctive devices to reduce procedural risks, such as cerebral embolic protection systems; and (5) a review of the currently available valve systems. Finally, we discuss (6) evolving strategies for lifetime management, including considerations around coronary re-access, prosthesis durability, and reintervention planning. These developments are shaping the future trajectory of TAVR as it continues to extend into broader and more complex clinical scenarios.

## 2. Current and Potential Future Indications

The first TAVR landmark trials demonstrated the non-inferiority of TAVR compared to SAVR in high surgical risk patients. Since then, a growing body of evidence has consistently demonstrated TAVR non-inferiority in all patient groups, including patients at very low surgical risk [5]. The PARTNER-3 trial randomized 1000 patients to either TAVR or SAVR and showed significantly lower composite outcomes of death, stroke, and readmission at 1 [6] and 2 years [7] in the TAVR group. This difference faded in the 5-year analysis [8], which suggests that the TAVR procedure derives benefit, especially because of lower procedural risk. PARTNER-3 was also the first trial that showed no increased paravalvular leaks when compared to SAVR [6]. The EVOLUT Low Risk trial similarly demonstrated the non-inferiority of TAVR using either CoreValve, Evolut R, or Evolut PRO with regard to a composite outcome of death and disabling stroke at 2 [9], 3 [10], and 4 years [11]. The recently published NOTION trial, which randomized 280 low-risk patients to TAVR vs. SAVR, reported no difference in a composite outcome of all-cause mortality, stroke, or myocardial infarction over 10 years. Interestingly, the trial reports a significantly lower rate of severe structural valve deterioration in favor of the TAVR group, demonstrating increased transcatheter heart valve (THV) resiliency over time [12].

In 2020, the American College of Cardiology/American Heart Association (ACC/AHA) published guidelines that recommend the use of transfemoral TAVR over SAVR for severe symptomatic AS in older patients (>80 years old) and recommend SAVR over TAVR for younger (<65 years old) patients. The Association for Cardio-Thoracic Surgery (ESC/EACTS) published similar guidelines in 2021 using the Society of Thoracic Surgeons risk score (STS-PROM) as a criterion to choose between SAVR and TAVR [13]. Still, clinical judgment remains key in selecting the right procedure for patients aged between 65 and 80 years old. Despite those guidelines, as the body of evidence to support the use of TAVR in younger and healthier patients grows, TAVR is now becoming the mainstream option proposed to young and healthy patients. A retrospective study reports that the proportion of patients < 65 years old in the US undergoing TAVR versus SAVR increased from 7.1% in 2013 to 54.7% in 2021 despite the lack of randomized controlled trials in this specific population [14]. Retrospective data suggests that while younger patients are undergoing TAVR, the ones that do usually present more comorbidities than their older counterparts, which probably influenced the heart team’s decision to choose a less invasive option [15]. Those numbers suggest an increase in the number of patients with BAV and AR undergoing TAVR. While the procedure has been used for those conditions in patients with prohibitive surgical risk, initial low-risk landmark trials have notably excluded patients with those indications because of technical and anatomical obstacles.

### 2.1. Bicuspid Aortic Valve

Compared to tricuspid aortic valves, BAVs often exhibit a more elliptical and asymmetric annulus, excessive leaflet calcification, and a higher incidence of calcified raphes that can interfere with valve expansion and anchoring. Furthermore, BAV is frequently associated with aortopathy, including the dilation of the ascending aorta, which may require surgical management and complicate the transcatheter approach [16]. These anatomical nuances can lead to increased rates of paravalvular leak, device malposition, and procedural complications.

Bicuspid aortic valve (BAV) anatomy poses unique challenges for TAVR due to its asymmetric calcification patterns, elliptical annulus, and associated aortopathy. While early experiences suggested that TAVR was technically feasible in selected BAV patients, even in the presence of heavily calcified raphes, outcomes have varied significantly depending on anatomical complexity [17]. In the NOTION-2 trial, which included a substantial number of low-risk patients with BAV, TAVR was associated with higher rates of non-disabling stroke (5.4% vs. 1.6%, *p* = 0.05) and mortality (though not statistically significant) compared with SAVR, likely reflecting the absence of strict anatomical exclusion criteria [18]. These findings underscore the importance of careful pre-procedural screening and anatomical assessment in BAV candidates for TAVR, as certain BAV phenotypes, such as Sievers type 2, remain less suitable.

Reassuringly, data from large contemporary registries such as the Society of Thoracic Surgeons (STS) and Transcatheter Valve Therapy (TVT) database have shown that stroke and mortality rates in selected BAV patients undergoing TAVR are now comparable to those observed in patients with tricuspid aortic valves [19]. These findings support the expanding role of TAVR in BAV disease, particularly in older patients or those at intermediate or high surgical risk, provided that anatomical suitability is carefully evaluated on a case-by-case basis. For instance, dilation of the aortic root in AR can increase the complexity of the procedure. There is considerable interest in this topic, and randomized controlled trials are currently being proposed and initiated to better define optimal patient selection and treatment strategies in BAV [20].

### 2.2. Aortic Regurgitation

While transcatheter aortic valve replacement (TAVR) has become well established in the treatment of aortic stenosis, its application in pure native aortic regurgitation (AR) presents unique technical and anatomical challenges that limit widespread adoption. Unlike stenotic valves, which are typically heavily calcified and thus provide a rigid and predictable annular landing zone, regurgitant valves often lack sufficient calcification, making it difficult to achieve secure anchoring of the transcatheter valve. This absence of calcium eliminates a critical stabilizing feature for prosthesis deployment, increasing the risk of valve malposition, migration, embolization, and significant paravalvular regurgitation [21].

Patients with aortic regurgitation (AR) often present with large, dilated annuli and ascending aortas, which can exceed the sizing limits of current transcatheter valves. This anatomical challenge reduces device stability and limits the effectiveness of oversizing, which may increase the risk of annular rupture or conduction disturbances. Undersizing, in contrast, raises the risk of paravalvular leak or embolization. The absence of calcification or a narrowed outflow tract, unlike in aortic stenosis, complicates valve positioning and axial alignment, making precise imaging critical for procedural planning.

These anatomical and hemodynamic complexities have historically relegated TAVR for AR to off-label use, primarily in inoperable or high-surgical-risk patients. However, device innovation is actively addressing these limitations. The ALIGN-AR trial, which evaluated the JenaValve (Irvine, CA, USA) Trilogy system, represents a significant milestone. This device employs a novel clip-based anchoring mechanism that attaches directly to the native aortic valve leaflets, bypassing the need for annular calcification or radial force for stability. In a cohort of 500 patients, the study reported a one-year all-cause mortality of 8.1%, with favorable hemodynamic performance and minimal residual regurgitation at follow-up, but a high rate (24%) of conduction system abnormalities requiring pacemaker implantation [22,23]. Notably, the system was specifically engineered for AR, which may signal a paradigm shift in the management of select patients with this condition. A recent 2025 meta-analysis, including 34 studies and 2162 patients comparing dedicated versus non-dedicated TAVR devices for AR, demonstrated superior procedural success and lower rates of all-cause mortality at 30 days and 1 year, pacemaker implantation, device migration, and paravalvular leak with dedicated devices, highlighting the importance of device selection in this challenging population [24].

Despite these promising findings, several unanswered questions remain. Long-term durability data are still lacking, particularly in younger patients or those with lower surgical risk, who may otherwise be eligible for surgical aortic valve replacement [16]. The presence of concomitant aortopathy, including annuloaortic ectasia or ascending aortic aneurysm, may still represent a contraindication for TAVR, necessitating comprehensive preoperative evaluation. Moreover, randomized controlled trials comparing TAVR to surgical options in the AR population are needed to establish safety, efficacy, and durability, and to better define optimal patient selection criteria [20].

### 2.3. Moderate AS

Emerging data suggest that earlier intervention with TAVR in moderate AS may benefit select high-risk patients, particularly those with heart failure and reduced ejection fraction. While the UNLOAD-TAVR trial, which compared TAVR to optimal medical therapy in such patients, was terminated early after enrolling only 178 of the planned 600 participants, it failed to demonstrate a significant advantage on a composite of death, disabling stroke, heart failure hospitalizations, or quality of life at one year [25]. Despite this, interest in the potential benefit of earlier valve intervention remains high. The ongoing EXPAND TAVR II Pivotal trial is evaluating transfemoral TAVR versus clinical surveillance in patients with moderate symptomatic AS and heart failure, with primary endpoints of all-cause mortality and heart failure hospitalizations [26]. Additionally, the PROGRESS trial is examining whether TAVR improves outcomes in patients with moderate AS who remain symptomatic despite medical therapy [27]. Collectively, these ongoing studies are expected to clarify the role of TAVR in moderate and asymptomatic AS and may help redefine traditional thresholds for intervention. These studies are expected to clarify the role of TAVR in moderate AS and may open the door to earlier treatment strategies.

## 3. Imaging and Pre-Procedural Planning

Adequate sizing of the prosthetic valve is crucial to minimize device embolization and paravalvular leak associated with undersized valves, but also to minimize the risk of altering the conduction system or causing an aortic root injury or annular rupture with an oversized prosthesis. Three-dimensional CT has become the gold standard to evaluate the anatomy prior to prosthesis selection and implantation [13,28,29]. This imaging modality offers the benefit of evaluating the different vascular access options and ruling out other potential relative contraindications, such as malignancy that could affect life expectancy. Three-dimensional CT also has the advantage of helping refine the prosthesis selection, especially for valve-in-valve (ViV) TAVR, and to ensure subsequent coronary access is possible [30]. The recent development of artificial intelligence (AI) has also impacted the pre-procedural planning. Several AI software have been developed to help with prosthesis selection and patient selection [31]. Platforms such as the DASI simulations (Montreal, QC, Canada) [32] and Materialise Mimics (Leuven, Belgium) [33] use AI computational modeling to simulate the deployment of the THV and can help predict the risks of the procedure, including paravalvular leak, annular rupture, and coronary obstruction. Other similar platforms, such as FEops (Ghent, Belgium) HEARTguide [34], allow for the 3D procedural planning of other structural cardiology interventions, such as percutaneous left atrial appendage occlusion, and will become more frequently used in the coming years. While more data is required, some systems have been validated when compared to expert clinicians to report anatomical measurement and suggest prosthesis size selection with concordance up to 88% [35].

## 4. Coronary Access

Coronary artery disease (CAD) is highly prevalent in patients with aortic stenosis. As such, another important aspect of pre-procedural evaluation is the possible need for further invasive diagnostic coronary imaging. The use of CT coronary angiography has demonstrated a good diagnostic yield to rule out CAD and has the advantage of being included in the pre-TAVR anatomical scan [36]. Nonetheless, sensitivity levels can vary from one center to another, and invasive coronary angiography may still be required. The question of coronary access after TAVR is a subject of great interest in the current literature. One of the first studies to quantify unfavorable coronary access was the RE-ACCESS trial, reporting up to 7.7% of coronary canulation failure, which mostly occurred exclusively with the Evolut THV [37]. A meta-analysis by Giacobbe et al. published in the *European Heart Journal* in 2024, which included 19 trials, comprising 1533 patients, reports that coronary access was possible in the majority of TAVR patients, with higher success rates for the left main coronary artery (LM) than for the right coronary artery (RCA) [38]. Balloon-expandable valves demonstrated high success rates for both ostia cannulation, with nearly 100% success for both LM and RCA, probably due to their intra-annular position. Among self-expandable supra-annular systems, the Evolut R/PRO showed successful access to the LM of 76.85% and to the RCA of 57.27% [38]. To facilitate coronary access of the supra-annular self-expandable systems, commissure alignment systems were developed. The Aligned-access study reported improved coronary access success with these systems for the supra-annular self-expandable valves (Evolut R and Acurate Neo), but still carried more risk of unsuccessful coronary access than the intra-annular balloon-expandable system (Sapien 3 by Edwards Lifesciences) [39]. Similar findings were also reported in the ALIGN TAVR study [40]. The RE-ACCESS 2 trial reported a 5.5% rate of coronary cannulation failure despite commissural alignment techniques with self-expandable systems, suggesting that ongoing refinements in these platforms are still needed to facilitate reliable coronary access [41]. The new Evolut Fx+ platform was designed to facilitate coronary access, with larger cells at the level of the coronary ostia. It has preliminarily been shown to be associated with 100% success of coronary canulation [42]. Other newer valves, such as the Navitor and the Acurate, are being designed with bigger, wider cells to facilitate coronary access. With ViV TAVR, the risk of coronary obstruction is a rare but feared complication, reported to occur in approximately 2.5% to 3.5% of procedures [43]. This risk is considerably higher than in native valve TAVR, primarily due to the displacement of the leaflets of the failed bioprosthesis toward the coronary ostia during implantation of the transcatheter valve. Predictors of coronary obstruction include a short virtual transcatheter valve-to-coronary (VTC) distance (<4 mm), low-lying coronary ostia, narrow sinuses of Valsalva, and supra-annular bioprostheses with externally mounted leaflets [44]. Higher THV implantation also increases the risk of unfavorable coronary access [45]. Detailed pre-procedural CT imaging is critical in evaluating these anatomic features and stratifying obstruction risk.

To mitigate coronary obstruction risk, several leaflet modification strategies have been developed. Initial preventive techniques included coronary protection using guidewires [46] and the chimney or snorkel stenting approach [47], where a stent is deployed from the coronary ostium to the aorta to preserve flow. Those have been associated with poor periprocedural outcomes, especially in bail-out settings [48]. Electrosurgery procedures lacerate failed leaflets through various mechanisms, such as BASILICA [49] (bioprosthetic aortic scallop intentional laceration to prevent coronary artery obstruction), UNICORN [50] (undermining iatrogenic coronary obstruction with radiofrequency needles), and LLAMACORN [51]. Each technique employs variations in energy delivery to split the bioprosthetic leaflets, allowing blood flow to the coronaries even after valve deployment. The main drawback of those techniques is the procedural complexity. It requires highly skilled and trained operators. To overcome this challenge, a newer device-based approach is the ShortCut system developed by Pi-Cardia (Rehovot, Israel), which is designed to mechanically cut through the leaflets of a degenerated bioprosthesis to prevent coronary obstruction. In the 2024 ShortCut study, published in the *European Heart Journal*, 60 patients with failed surgical bioprostheses underwent ViV TAVR using the device. The study reported a freedom from coronary obstruction at 30 and 90 days of 95%, with favorable safety and feasibility outcomes [52].

While this is a rapidly evolving field with growing interest, direct comparisons between different coronary protection and leaflet modification techniques remain limited. A comparative study between chimney stenting and BASILICA showed similar procedural and one-year clinical outcomes [53], although further trials are needed to evaluate their relative efficacy, safety, and technical complexity. As ViV TAVR procedures continue to increase, especially in younger populations with prior bioprostheses, careful pre-procedural planning and the development of dedicated tools and techniques will be essential to mitigate procedural risks and optimize long-term outcomes.

## 5. Cerebrovascular Events Prevention

TAVR is associated with an increased cerebrovascular event (CVE) risk up to 2 years after the procedure when compared to the general population [54]. A recent retrospective study reported the risk of CVE at 30 days post-TAVR at 3%, with 69% of those occurring in the first 48 hours [54]. In another registry study of 176,316 patients undergoing TAVR between 2011 and 2019, the overall in-hospital stroke rate decreased slightly from 2.1% in the early part of the study to 1.6% in 2019 [55]. The mechanism by which TAVR increases the risk of CVE is believed to be related to calcium or plaque emboli. To prevent early periprocedural CVEs, cerebral embolism protection systems, such as Protembo [56], Emboliner [57], Emblok [58], and Sentinel [59], were designed. They are devices placed in the aortic arch or inside the carotid during TAVR to prevent embolization to the cerebral circulation. Despite initial interest, the early randomized controlled trials using those systems failed to demonstrate a decrease in post-procedural CVEs [59]. Retrospective studies also failed to demonstrate an independent benefit of these systems concerning strokes, but their use was associated with a lower risk of major stroke in a multivariate model [60]. The recently published randomized controlled trial, BHF PROTECT-TAVI trial, also failed to demonstrate a benefit from the routine use of a cerebral embolic protection device [61].

## 6. The Different Valve Systems

Both surgical valves and transcatheter valves (THVs) can, unfortunately, fail over time. As new valve models were developed, they became more resilient with better hemodynamic profiles. The most common THV systems used today are classified according to their opening mechanism.

Self-expandable valves include Medtronic’s (Minneapolis, MN, USA) Evolut family, Boston Scientific’s (Marlborough, MA, USA) Acurate neo, Abbott’s (Abbott Park, IL, USA) Navitor, JenaValve Technology’s Trilogy system, and SMT’s (Sahajanand Medical Technologies) (Surat, Gujarat, India) Hydra valve. Recent models, such as Medtronic’s Evolut FX+, include modifications to the nitinol frame designed to optimize commissural alignment and coronary access. Following post-market concerns related to valve thrombosis and embolization risk, Abbott’s Portico model was voluntarily withdrawn. A revised model called Navitor, addressing these concerns, has since been reintroduced and is currently available [62].

Boston Scientific’s Acurate neo valve, another self-expandable option, was found to be inferior to other platforms in early studies. The SCOPE 1 trial demonstrated that Acurate neo had worse early safety and device performance compared to the balloon-expandable Sapien 3 valve, particularly in terms of paravalvular regurgitation and early mortality [63]. Similarly, SCOPE 2 compared Acurate neo with Evolut and revealed higher all-cause mortality and stroke at 30 days in the Acurate neo group [64]. More recently, the ACURATE IDE trial compared the updated Acurate Neo2 valve with Sapien 3 and failed to demonstrate non-inferiority in the composite primary endpoint of death or stroke at 30 days, further reinforcing the need for careful device selection [65]. Acurate Neo2 has recently been discontinued by Boston Scientific.

JenaValve’s Trilogy system, developed for aortic regurgitation, is based on a native valve’s cusp grasping mechanism that decreases paravalvular leaks.

Balloon-expandable valves include Edwards Lifesciences’ (Irvine, CA, USA) Sapien and Meril Life Sciences’ (Vapi, Gujarat, India) MyVal. While the early literature seemed to favor the use of balloon-expandable systems [66], recent trials have shown that self-expandable and balloon-expandable valve systems are equivalent in terms of all-cause mortality, stroke, permanent pacemaker implantation, and paravalvular leaks [67,68,69].

The latest balloon-expandable SAPIEN 3 Ultra (Ultra 3) transcatheter heart valve (THV) is the first THV to utilize Resilia bovine pericardial tissue—a proprietary anticalcification process originally developed for surgical bioprostheses. In the COMMENCE trial of surgical Inspiris Resilia valves (*n* = 689), Resilia tissue demonstrated a marked reduction in structural valve deterioration-related hemodynamic valve deterioration (HVD) at five years, with grade ≥ 2 HVD occurring in only 1.8% of the patients versus 3.5% in a contemporary surgical control arm (one-sided 95% lower-bound hazard ratio 1.15; *p* = 0.03) [70]. In vitro accelerated wear testing simulating 2 billion cycles (equivalent to ~50 years) showed hemodynamics and peak flow velocities without evidence of gross damage or pannus formation [71]. Early real-world registry data for the fifth-generation SAPIEN 3 Ultra RESILIA THV report a mean residual gradient of 10 ± 3 mmHg, effective orifice area  >  2.0 cm^2^, and no moderate or severe structural valve deterioration at one year [72]. Ongoing randomized and observational studies are now assessing whether these promising surgical valve findings translate into superior long-term durability and hemodynamic performance in the transcatheter platform, including head-to-head comparisons with next-generation SAPIEN X4 devices.

All the aforementioned valve systems are made with either porcine or bovine pericardium pieces sutured together to create the commissures. Recently, a new model of valve was developed by Anteris Technologies (Brisbane, QLD, Australia) by molding a single piece of tissue into the shape of a native aortic valve, possibly creating a larger orifice area. Preliminary results of this biosimilar prosthesis are encouraging, with performance sustained at 1 year and restoration of near-normal flow dynamics [73]. Similarly, polymer valves have shown promising data, potentially offering longer durability, better biocompatibility, and reduced cost [74]. The Polynova (Los Angeles, CA, USA) [75] and Tria LP by Foldax (Salt Lake City, UT, USA) [76] have proven safety in trials, but no trials clinically comparing polymeric THV to biologic THV have neither completed nor ongoing.

## 7. Lifetime Management

While initial concerns regarding the complications, especially related to vascular access, CVEs, and conduction abnormalities, were daunting, the development and improvement of TAVR have made it a safe and efficient alternative to SAVR for a large proportion of the population with an indication for AVR. With these improvements, as the cost of complications associated with TAVR continues to decrease, the optimal timing of AVR may gradually shift toward less severe and less symptomatic AS. This has been studied in many recently published trials. The EARLY-TAVR trial randomly assigned patients with asymptomatic severe AS to either clinical surveillance or TAVR and demonstrated a decrease in the composite outcome of death, stroke, or unplanned hospitalization for CV causes in favor of TAVR [77]. Supporting this, a meta-analysis including 1427 patients in four randomized controlled trials, published in JACC earlier this year, confirmed that the earlier valve replacement compared with clinical surveillance was associated with a significant reduction in unplanned CV or HF hospitalization and stroke, with no difference in CV and all-cause death [78]. Other trials are currently ongoing, such as the EASY-AS trial, which is investigating the efficacy of early aortic valve intervention in severe asymptomatic AS patients with preserved left ventricular function [79]. Parallel to this, the FDA has recently approved TAVR in selected asymptomatic patients with severe AS based on emerging data, a decision that may signal a paradigm shift toward earlier treatment even before the onset of symptoms [80].

As TAVR expands into younger populations, unique challenges emerge, chiefly related to valve durability, lifetime management, and coronary re-access. Younger patients (< 65 years) have longer life expectancy and are more likely to require multiple valve interventions over their lifetime. The recently published DEDICATE trial, a randomized, non-inferiority study enrolling low-risk patients aged 60–75 years, compared contemporary balloon-expandable TAVR prostheses with surgical bioprosthetic AVR, with coprimary endpoints of composite death or disabling stroke at two years and hemodynamic valve performance at five years [81]. Early results from DEDICATE demonstrated comparable rates of all-cause mortality and disabling stroke at 24 months between TAVR and SAVR arms, with a trend toward lower perioperative morbidity in the TAVR cohort [81]. A companion rationale and design paper outlines key anatomical inclusion criteria, such as annular dimensions permitting valve-in-valve repeatability, and prespecified lifetime management strategies, including provisions for coronary access planning and sequential TAVR procedures [82]. These data will be critical in informing heart-team decision-making for younger candidates, balancing the benefits of a less invasive approach against the unknown long-term durability of transcatheter bioprostheses.

**Figure 1:**
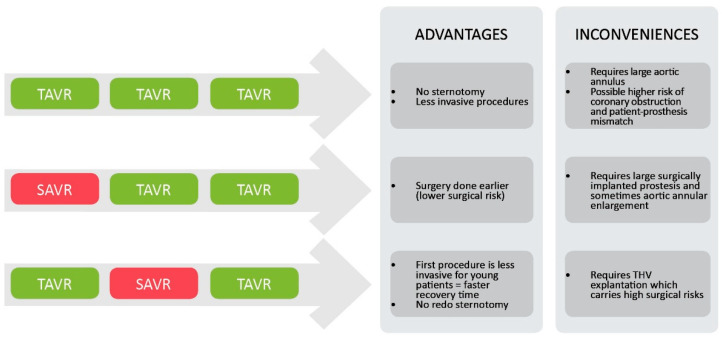
Lifetime management strategies for patients undergoing several aortic valve replacement procedures.

As TAVR is used more frequently, especially in younger patients, equipoise persists as to the best strategy for the lifetime management of patients requiring several subsequent AVR. As TAVR has not been systematically tested in patients < 65 years old, unanswered questions of durability, coronary access, and risk of redo TAVR remain. For example, in current clinical practice, a young, low-surgical-risk patient requiring AVR may undergo SAVR first, then TAVR-in-SAVR if the prosthesis degenerates. The risk of performing TAVR as the first procedure raises several concerns regarding subsequent valve interventions. As the valve degenerates, the subsequent prosthesis will need to be of a smaller size, further increasing the risk of patient–prosthesis mismatch. For very young patients for whom we may consider three interventions, there are three possible scenarios, as reported by Yerasi et al. in JACC in 2021: (1) TAVR-TAVR-TAVR, (2) SAVR-TAVR-TAVR, and (3) TAVR-SAVR-TAVR [83]. See Figure 1. An only-TAVR strategy (TAVR-TAVR-TAVR) might be possible for patients with a suitable anatomy, which consists of a very large valvular annulus and wide sinuses of Valsalva to prevent coronary ostia obstruction, using intra-annular balloon-expandable systems. A SAVR-first strategy allows for the surgery to be performed at a younger age and hence carries a lot less surgical risk and is associated with lower morbidity and mortality. The downside of this approach is the need for a large enough surgically implanted valve to allow for two subsequent TAVR. A TAVR-first, SAVR-second (and TAVR third if necessary) strategy has the benefit of offering young, working-age patients a minimally invasive procedure first. The subsequent SAVR is realized on a never-opened-before chest, though it often requires concurrent aortic root repair with the explantation of the first prosthesis. If needed, a third procedure, TAVR-in-SAVR, can be performed with relative safety. The main drawback of this approach is the need for aortic THV explantation, which is still a relatively uncommon procedure and carries very high risks [84,85].

As TAVR becomes more frequently used, the lifetime management of AS requiring AVR becomes more complex. Several strategies have been described, and this remains an area of great interest in research. As of today, no “one-size-fits-all” pathway can be proposed, given the different etiologies of AS and the concurrent comorbidities [86].

## 8. Conclusions

TAVR has transformed the treatment of aortic stenosis, initially for high-risk patients but now increasingly used in intermediate- and low-risk populations. Evidence from trials and real-world data highlights its benefits in survival, recovery, and quality of life, supporting its role as a cornerstone of modern cardiac care. As indications broaden, recent advancements in valve technology may improve prosthesis durability and help address the challenges of lifetime valve management. While issues like long-term outcomes, complications, and equitable access remain, TAVR continues to shift the clinical paradigm toward earlier, less invasive intervention with promising outcomes.

## Abbreviations

TAVR	Transcatheter Aortic Valve Replacement
AS	Aortic Stenosis
SAVR	Surgical Aortic Valve Replacement
AR	Aortic Regurgitation
BAV	Bicuspid Aortic Valve
CT	Computed Tomography
CAD	Coronary Artery Disease
CVE	Cerebrovascular Events
STS	Society of Thoracic Surgeons
TVT	Transcatheter Valve Therapy
THV	Transcatheter Heart Valve
ACC	American College of Cardiology
AHA	American Heart Association
ESC	European Society of Cardiology
EACTS	European Association for Cardio-Thoracic Surgery
STS-PROM	Society of Thoracic Surgeons Predicted Risk of Mortality
ViV	Valve-in-Valve
AI	Artificial Intelligence
LM	Left Main (coronary artery)
RCA	Right Coronary Artery
VTC	Virtual Transcatheter Valve-to-Coronary
BASILICA	Bioprosthetic Aortic Scallop Intentional Laceration to prevent Coronary Artery obstruction
UNICORN	Undermining Iatrogenic Coronary Obstruction with Radiofrequency Needle
LLAMACORN	Leaflet Laceration with Balloon Mediated Annihilation to prevent Coronary Obstruction with Radiofrequency Needle
FDA	Food and Drug Administration
CV	Cardiovascular
HF	Heart Failure
HVD	Hemodynamic Valve Deterioration
